# Supplementary material for: The causal role of circulating inflammatory markers in osteoporosis: a bidirectional Mendelian randomized study
Source: Front Immunol. 2024 Jul 18;15:1412298. doi: 10.3389/fimmu.2024.1412298 (PMC11291241; doi:10.3389/fimmu.2024.1412298)
Supplement: Supplementary file 1 [file DataSheet_1.zip › supplementary material/OSTEOPOROSIS-CIM/GCST90274798.tsv.gz_forest.pdf]

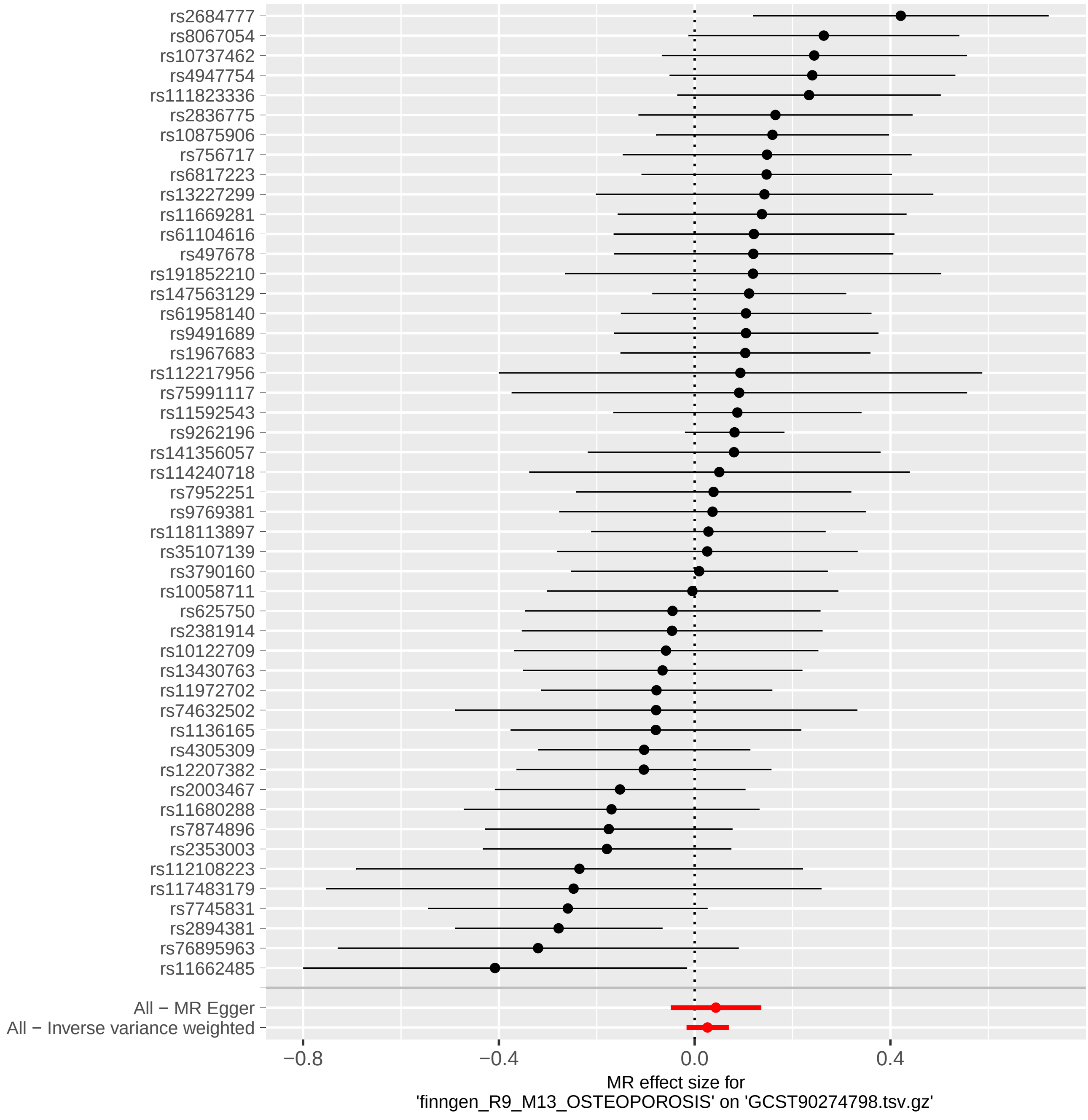
rs2684777
rs8067054
rs10737462
rs4947754
rs111823336
rs2836775
rs10875906
rs756717
rs6817223
rs13227299
rs11669281
rs61104616
rs497678
rs191852210
rs147563129
rs61958140
rs9491689
rs1967683
rs112217956
rs75991117
rs11592543
rs9262196
rs141356057
rs114240718
rs7952251
rs9769381
rs118113897
rs35107139
rs3790160
rs10058711
rs625750
rs2381914
rs10122709
rs13430763
rs11972702
rs74632502
rs1136165
rs4305309
rs12207382
rs2003467
rs11680288
rs7874896
rs2353003
rs112108223
rs117483179
rs7745831
rs2894381
rs76895963
rs11662485
All − MR Egger
All − Inverse variance weighted
−0.8
−0.4
0.0
0.4
MR effect size for
'finngen_R9_M13_OSTEOPOROSIS' on 'GCST90274798.tsv.gz'
